# 

**Published:** 1931

**Authors:** 


### The Bristol
### Medico-Chirurgical Journal

A JOURNAL OF THE MEDICAL SCIENCES FOR THE
WEST OF ENGLAND AND SOUTH WALES.

PUBLISHED UNDER THE AUSPICES OF
*THE BRISTOL MEDICO-CHIRURGICAL SOCIETY.*

EDITOR:
J. A. NIXON, C.M.G., M.D., F.R.C.P.

WITH WHOM ARE ASSOCIATED
E. W. HEY GROVES, M.S., F.R.C.S., B.Sc.
E. WATSON-WILLIAMS, M.C., CH.M., F.R.C.S.
A. E. ILES, O.B.E., M.B., B.S., F.R.C.S.
CAREY F. COOMBS, M.D., F.R.C.P., ASSISTANT EDITOR.

EDITORIAL SECRETARY:
A. L. FLEMMING. M.B., CH.B.

---

"Scire est nescire, nisi id me

Scire alius sciret."

---

*VOL. XLVIII.*

BRISTOL: J. W. ARROWSMITH LTD.

LONDON: J. W. ARROWSMITH (LONDON) LTD.

R31
.B8

### Contents of Vol. XLVIII.

Page

The Relation of Gynæcology to the Glands of Internal Secretion. By D. C. Rayner, Ch.M., F.R.C.S. .. .. .. .. 1

Ectopic Pregnancy. By R. S. S. Statham, M.D., Ch.M., F.C.O.G., and H. L. Shepherd, M.B., Ch.M., M.C.O.G. .. .. .. .. 15

Necrosis of the Myocardium. By Herbert Rogers, M.B., Ch.B. .. 35

Congenital Heart Disease as seen in Elementary School Children. By C. Bruce Perry, M.D., M.R.C.P. .. .. .. .. 41

The Differential Diagnosis of Primary Iritis in the Early Stages. By E. R. Chambers, F.R.C.S. .. .. .. .. .. .. 51

Some Reminiscences and a Forecast. By Sir Ewen J. Maclean, M.D., D.Sc. (Hon.), F.R.C.P., F.A.C.S. (Hon.), F.R.S.E. .. .. 83

The Modern Outlook on Mental Health. By L. G. Brock, C.B. .. 97

Some Intestinal Malformations and their Clinical Significance. By Arthur L. Taylor, M.D. .. .. .. .. .. .. 113

Some Disorders of Movement and their Mechanism. By H. H. Carleton, M.D., M.R.C.P. .. .. .. .. .. .. 135

Strangulated Hernia. By Noël Kemm, M.B., B.S., F.R.C.S. .. 151

A Review of Seven Hundred Cases of Acute Appendicitis. By Hubert Chitty, M.S., F.R.C.S... .. .. .. .. .. .. 167

The Work of the University Centre of Cardiac Research, 1927–31. By Carey F. Coombs, M.D., F.R.C.P. .. .. .. .. 179

Sterilization of the Unfit. By H. C. Bristowe, M.D. .. .. 189

Tetany following Thyroid Operations. By Duncan Wood, F.R.C.S. 205

A Case of Congenital Obstruction of the Bile-Duct. By J. A. Birrell, M.D., M.R.C.P., and A. Wilfrid Adams, M.S., F.R.C.S. .. 215

The Long Fox Memorial Lecture: The Partnership between Anæsthesia and Surgery. By Arthur L. Flemming, M.B., Ch.B., L.R.C.P., M.R.C.S. .. .. .. .. .. .. .. .. .. 233

Silicosis :—

I.—Administrative and Clinical. By J. A. Nixon, C.M.G., M.D., F.R.C.P. .. .. .. .. .. .. 253

II.—Radiology and Pathology. By G. B. Bush, M.B., Ch.B., D.M.R.E. .. .. .. .. .. .. .. 259

Congenital Abnormalities of the External Ear. By E. Watson-Williams, M.C. .. .. .. .. .. .. .. 273

Hæmaturia in the Pre-Eruptive Stage of Measles. By F. G. Jenkins, M.B., Ch.B. .. .. .. .. .. .. .. 275

Cyanosis in the Fatal Broncho-Pneumonia of Influenza. By Frank E. Fletcher .. .. .. .. .. .. .. .. 277

Reviews of Books .. .. .. .. .. .. 57, 155, 219, 283

Editorial Notes .. .. .. .. .. .. 65, 159, 223, 289

Obituaries .. .. .. .. .. .. .. .. 65, 226

Meetings of Societies .. .. .. .. .. .. 67, 162, 292

The Medical Library of the University of Bristol 70, 164, 229, 296

Local Medical Notes .. .. .. .. .. 73, 165, 231, 299

### The Medical Library of the University of Bristol,

WITH WHICH IS INCORPORATED

**The Library of the Bristol Medico-Chirurgical Society.**

*The following donations have been received since the publication of the List in December,* 1930.

February, 1931.

| | | |
|---|---|---|
| Association of American Physicians (1) | .. | 1 volume |
| Dr. W. S. Bainbridge (2) | .. .. .. | 1 ,, |
| Carnegie Institution (3) | .. .. .. | 1 ,, |
| Michell Clarke Fund (4) | .. .. .. | 41 volumes |
| Professor E. W. Hey Groves (5) | .. .. | 9 ,, |
| Dr. M. Nierenstein (6) .. | .. .. .. | 1 volume |
| Philadelphia General Hospital (7) | .. .. | 1 ,, |
| Rockefeller Foundation (8) | .. .. .. | 1 ,, |
| Smithsonian Institution (9) | .. .. .. | 4 volumes |
| C. H. Walker, Esq. (10) | .. .. .. | 4 ,, |
| Wilmer Institution (11) | .. .. .. | 1 volume |

Unbound periodicals have also been received from Professor Hey Groves.

### THE ONE HUNDRED AND THIRTY-FIFTH LIST OF BOOKS.

The figures in round brackets refer to the figures after the names of the donors. The books to which no such figures are attached have either been bought from the Library Fund or received through the *Journal.*

**Ablett, R.** .. .. .. *Course in Physics for Medical and Dental Students* .. .. .. .. .. .. .. .. .. 1930
**Aschoff, L.** .. .. *Der Appendicitische Anfall* .. .. .. .. (5) 1930
**Ashton, G.** .. .. *Royal Army Medical Corps Aids to Dispensing* 1930
**Bainbridge, W. S.** .. *Consideration of the Psychic Factors in Surgical Diagnosis and Procedure* .. .. .. .. (2) 1930
**Beckman, H.** .. .. *Treatment in General Practice* .. .. (4) 1930
**Boycott, A. E.** .. .. *Transition from Live to Dead* .. .. (9) 1930
**Brockman, E. P.** .. *Congenital Clubfoot* .. .. .. .. .. .. .. .. 1930
**Brown, W. Langdon, and Hilton, R.** *Physiological Principles in Treatment* .. .. .. .. .. 6th Ed. (4) 1930
**Cathcart, G. C.** .. *Treatment of Chronic Deafness by the Electro-phonoide Method of Zünd-Burguet* 2nd Ed. 1931
**Cavendish, H.** .. .. *Rathbone Place Water* .. .. .. .. .. .. 1766
*Cinchona Tercentenary Celebration and Exhibition* .. .. .. .. .. 1930
*Collected Reprints from the Wilmer Ophthalmological Institution of the Johns Hopkins University and Hospital.* Vol. I. 1925 to 1929 .. .. .. .. .. .. .. .. .. .. .. .. .. .. (11) 1929
**Collins, E. T.** .. .. *History and Traditions of the Moorfields Eye Hospital* .. .. .. .. .. .. .. .. .. 1929
*Concilium Ophthalmologicum,* xiii, 1929 *Hollanda* .. .. 4 vols. (10) 1930
**Cumberbatch, E. P.** *Essentials of Medical Electricity* 6th Ed. (4) 1929
**Curgenven, J. S.** .. *Child's Diet* .. .. .. .. .. .. 3rd Ed. 1929
**Davidson, L. S. P., and Gulland, G. L.** *Pernicious Anæmia* .. (4) 1930
**Davies, H. M.** .. .. *Surgery of the Lung and Pleura* .. .. (4) 1930
**Delafield, F., and Prudden, T. M.** *Text-book of Pathology* 14th Ed. (4) 1928
**Donnan, F. G.** .. .. *Mystery of Life* .. .. .. .. .. .. .. (9)
**Douthwaite, A. H.** .. *Injection Treatment of Varicose Veins* 5th Ed. 1929
**Douthwaite, A. H.** .. *Treatment of Asthma* .. .. .. .. .. .. 1930
**Drummond, J. C., and Hilditch, T. P.** *Relative Values of Cod Liver Oil from Various Sources* .. .. .. .. .. (6) 1930
**Evans, C. L.** .. .. *Recent Advances in Physiology* 4th Ed. (4) 1930
**Eyre, H.** .. .. .. *Brief Account of the Holt Waters* .. .. 1743
**Ferrée, C. J.** .. .. *Soya Bean and the New Soya Flour* .. .. 1929
**Flexner, S.** .. .. *Hideyo Noguchi* .. .. .. .. .. .. .. (9) 1930
**Fothergill, A.** .. .. *Inquiry into Nature and Qualities of Cheltenham Waters* .. .. .. .. .. .. .. .. .. 1788
**Géraudel, É.** .. .. *Mechanism of the Heart and its Anomalies* .. 1930
**Gray, H.** .. .. .. *Anatomy* .. .. .. .. .. 24th Ed. (4) 1930
**Gruner, O. C.** .. .. *Canon of Medicine of Avicenna* .. .. (4) 1930
**Gunn, T. A.** .. .. *Introduction to Pharmacology and Therapeutics* 2nd Ed. (4) 1931
**Halliburton, W. D., Hewitt, J. A., and Robson, W.** *Essentials of Chemical Physiology* .. .. 12th Ed. (4) 1929

**Haslam** .. .. .. *Recent Advances in Preventive Medicine* (4) 1930
**Helmont, J. B. von** *Oriatrike or Physick Refined* .. .. .. .. .. 1662
**Hess, A. F.** .. .. *Rickets* .. .. .. .. .. .. .. .. .. (4) 1930
**Hofmann, A. W.** .. *Harrogate and its Resources* .. .. .. .. .. 1854
**Hughes, E.** .. .. *Seasonal Variation in Man* .. .. .. .. .. 1931
**Jameson, W. W., and Parkinson, G. S.** *A Synopsis of Hygiene* 3rd Ed. (4) 1930
**Jellett, H.** .. .. .. *Short Practice of Midwifery* .. 10th Ed. (4) 1930
**Jellett, H., and Madill, D. G.** *Manual of Midwifery* .. 4th Ed. (4) 1929
**Jones, Sir R.** .. .. *Injuries to Joints* .. .. .. 3rd Ed. (5) [1930]
**Kelly** .. .. .. .. *Directory of Bristol and Suburbs for* 1931 .. [1931]
**King, J.** .. .. .. *Essay on Hot and Cold Bathing* .. .. .. .. 1737
**Lawrence, R. D.** .. *Diabetic A.B.C.* .. .. .. .. .. .. (4) 1930
**Lawrence, R. D.** .. *Diabetic Life* .. .. .. .. 5th Ed. (4) 1930
*Lehrbuch der Chirurgie* .. .. .. .. .. .. .. .. Bds. 1 and 2 (5) 1930
**Liddell, E. G. T., and Sherrington, Sir C.** *Mammalian Physiology* 2nd Ed. (4) 1929
**McMurrich, J. P.** .. *Leonardo Da Vinci* .. .. .. .. .. (3) 1931
**Massie, G.** .. .. .. *Surgical Anatomy* .. .. .. .. .. .. (4) 1928
*Methods and Problems of Medical Education* .. .. .. .. .. .. .. 1930
*Narrative of the Efficacy of the Bath Waters* .. .. .. .. .. .. 1877
**Osler, Sir William** .. *Principles and Practice of Medicine* 11th Ed. (4) 1930
**Parsons, Sir J. H.** .. *Diseases of the Eye* .. .. .. .. .. .. (4) 1930
**Park, W. H., and Williams, A. W.** *Pathogenic Micro-organisms* 9th Ed. (4) 1929
**Perry, W. J.** .. .. *Gods and Men* .. .. .. .. .. .. .. (4)
**Piney, A., and Wyard, S.** *Clinical Atlas of Blood Diseases* .. .. (4) 1930
**Rea, R. L.** .. .. *Affections of the Eye in General Practice* .. 1930
**Rogers, Sir L.** .. .. *Recent Advances in Tropical Medicine* 2nd Ed. (4) 1929
**Rose and Carless** .. *Manual of Surgery* .. .. .. 13th Ed. (5) 1930
**Schäfer, Sir E. S.** .. *Essentials of Histology* .. .. 12th Ed. (4) 1929
**Short, T.** .. .. .. *Mineral Waters of Derbyshire, Lincolnshire and Yorkshire* .. .. .. .. .. .. .. .. 1734
**Sobotta, J.** .. .. *Atlas of Human Anatomy* Ed. from 7th German Ed. .. .. .. 3 vols. (4) 1923–29
**Spenser, H. R.** .. .. *Medicine in the Days of Shakespeare* .. (4) 1929
**Stewart, C. P., and Dunlop, D. M.** *Clinical Chemistry in Practical Medicine* .. .. .. .. .. .. (4) 1930
**Stopford, J. S. B.** .. *Sensation and the Sensory Pathway* .. (4) 1930
**Stroganoff, W.** .. *Improved Prophylactic Method in Treatment of Eclampsia* .. .. .. .. .. .. (4) 1930
*Studies on the Diagnosis and Nature of Cancer* .. .. .. .. .. ..
*System of Bacteriology* .. .. .. .. .. .. .. .. Vols, 1–5, 7 1930
**Tandler, J.** .. .. *Lehrbuch d. Systematischen Anatomie* 4 Bande (4) 1923–1929
**Taylor** .. .. .. *Practice of Medicine* .. .. 14th Ed. (4) 1930
**Tenenbaum, J.** .. .. *Riddle of Sex* .. .. .. .. .. .. .. .. 1930
**Ten Teachers** .. .. *Diseases of Women* .. .. .. 4th Ed. (4) 1930
**Topley, W. W. C., and Wilson, G. S.** *Principles of Bacteriology and Immunity* .. .. .. .. 2 vols. (4) 1929

| | | |
|---|---|---|
| | *Treatise on Mineral Waters* .. .. .. .. .. .. .. .. .. .. | 1766 |
| **Treves, Sir F.** .. .. | *Student's Handbook of Surgical Operations* 5th Ed. | 1930 |
| **Troup, W. A.** .. .. | *Therapeutic Uses of Infra-red Rays* .. .. | 1930 |
| **Wakeley, C. P. G., and Buxton, St. J. D.** | *Surgical Pathology* .. .. | 1929 |
| **Waterson, D.** .. .. | *Anatomy in the Living Model* .. .. .. | 1931 |
| **Wear, A.** .. .. .. | *A.B.C. of Child Health* .. .. .. .. .. .. | 1930 |
| **Williams, G.** .. .. | *Minor Surgery and Bandaging* 20th Ed. (5) | 1930 |
| **Williams, S.** .. .. | *History of Road Water in Wiltshire* .. .. | 1731 |
| **Wynter, J.** .. .. | *Essay on Chronical Diseases* .. .. 2nd Ed. | 1827 |

TRANSACTIONS, REPORTS, JOURNALS, ETC.

| | |
|---|---|
| Association of American Physicians. Transactions. Vol. XLV (1) | 1930 |
| Imperial Cancer Research Fund. Ninth Scientific Report .. (5) | 1930 |
| Medical Directory, 1931 .. .. .. .. .. .. .. .. .. .. .. .. | 1931 |
| Medical Society. Transactions. Vol. LIII. .. .. .. .. .. .. | 1930 |
| Philadelphia General Hospital Reports. Vol. XI. .. .. .. .. .. | 1931 |
| St. Bartholomew's Hospital Reports. Vol. LXIII and Supplement | 1930 |
| Progressive Medicine. December, 1930 .. .. .. .. .. .. .. .. | 1930 |
| Smithsonian Institution. Annual Report for 1929 .. .. .. (9) | 1930 |
| Surgical Clinics of North America. December, 1930 .. .. .. | 1930 |

### Local Medical Notes

EXAMINATION RESULTS.

**University of Bristol.**—Students of the University have recently passed the following examinations :—

M.D.—Albert Edwin Hayward Pinch.

M.B., CH.B. — *Second Examination* (*Part I.*). *Pass :* Kathleen G. Brimelow, F. C. Collingwood, Theodora M. Crabtree, J. G. Field, P. N. Heron, A. E. Jowett, S. D. Loxton, M. A. Nicholson, B. Ridgway, A. W. Woolley. *In Organic Chemistry* (*Completing Examination*) *:* T. R. V. Gurney. *In Organic Chemistry only :* G. W. Vowles.

M.B., CH.B.—*Second Examination* (*Part II.*). *Pass :* Grace J. V. Ball, Dorothy E. Barber, A. G. W. Branch (with distinction in Anatomy), J. R. G. Damrel, Rosalind M. S. Derham, N. H. Greenberg, J. N. Heales, A. J. Matheson, A. C. Molden (with distinction in Anatomy), Marion L. Tryon, F. L. Wollaston.

CONJOINT BOARD.—M.R.C.S., L.R.C.P.—*In Chemistry:* P. M. W. Martin, J. A. Cochrane. *In Physics:* P. M. W. Martin. *In Elementary Biology:* J. H. Bulleid. *In Anatomy and Physiology:* G. M. Denning. *In Pathology:* R. G. Wilbond. *In Medicine:* J. L. S. Coulter,* J. A. Kersley,* Anne E. Williams-James,* L. R. Jordan,* W. A. F. Taylor.* *In Surgery:* Anne E. Williams-James,* L. R. Jordan,* W. A. F. Taylor,* R. V. C. Richards, C. J. N. Davis. *In Midwifery:* H. D. Pyke,* C. J. N. Davis.

B.D.S.—*Second Examination. Pass (in Dental Anatomy only):* J. W. E. Snawdon.

L.D.S., R.C.S.—*First Professional Examination. Part I. (Dental Mechanics):* Beryl M. Davies, R. H. Green, S. A, McCandlish, P. J. F. Shepherd, H. W. South, W. N. Trays, T. H. Williams. *Part III. (a) (Anatomy and Physiology):* T. H. Williams, B. H. W. Baker, G. L. R. Welch, D. Gent. *Part III. (b) (Dental Anatomy):* J. S. Cochrane, A. C. Davies, O. F. Harley, A. E. Hawkins, N. E. Miles, H. F. Vere.

L.D.S.—*First Professional Examination. Pass (in Dental Anatomy, Completing Examination):* I. R. R. Eskell, R. L. Royal.

L.D.S., R.C.S.—*Final Examination. Part I.:* W. U. Harwood. *Part II.:* G. E. Chadd, E. R. Dowland, W. A. Duly, E. S. Edwards, J. J. Hinton, D. R. Jones, R. E. Morgan, J. K. Noot, A. M. Watson.

L.D.S., R.C.S.—*Final Examination.* G. H. Dakers.*

* Qualified.

---

**Royal College of Physicians, London.**—The Goulstonian Lectures were delivered at the College by Dr. Macdonald Critchley on 10th, 12th and 17th March. The subject was "The Neurology of Old Age."

**Committee's Gold and Silver Medals.**—These medals have been awarded to Mr. J. J. J. Giraldi and to Mr. G. F. Langley respectively.

**Long Fox Memorial Lecture.**—This will be delivered in the Autumn by Mr A. L. Flemming, whose subject will be "The Partnership between Anæsthesia and Surgery."

**Radcliffe Prize for the Furtherance of Medical Science.**—This prize has been awarded by the Master and Fellows of University College, Oxford, to Dr. W. H. Bradley, Medical Officer to Downside School.

### Bristol Medico-Chirurgical Society.

### President.

D. C. RAYNER, Ch.M. Bristol.

### President-Elect.

J. R. CHARLES, M,A., M.D,. B.C. Cantab.

### Committee.

*Ex-Officio* { J. A. NIXON, *C.M.G.*, B.A., M.D. Cantab. (Editor of *Journal*).
G. PARKER, M.A., M.D. Cantab. (Hon. Librarian).

*Elected.*

W. K. WILLS, *O.B.E.*, M.A., M.B., B.Ch. Cantab. } Ex-Presidents.
C. FERRIER WALTERS, F.R.C.S. }
DUNCAN WOOD, F.R.C.S.
R. S. S. STATHAM, *O.B.E.*, M.D., M.Ch. Bristol.
H. CHITTY, M.S. Lond., F.R.C.S.
A. R. SHORT, M.D. Lond.
E. W. HEY GROVES, M.S., F.R.C.S., B.Sc. Lond.
H. G. KYLE, M.A., M.D., B.Ch. Oxon.
W. P. KENNEDY, M.D. Dub.

### Honorary Secretary.

W. A. JACKMAN, M.B., F.R.C.S. Ed.

### Honorary Treasurer.

A. E. ILES, *O.B.E.*, M.B., B.S. Lond., F.R.C.S.

### University Library Sub-Committee.

G. PARKER, M.A., M.D. Cantab. A. R. SHORT, B.Sc., M.D., B.S. Lond.
J. O. SYMES, M.D. Lond.

Medical Practitioners wishing to join the Society are requested to communicate with the Hon. Secretary, Mr. W. A. Jackman, 15 Mortimer Road, Clifton. The Annual Subscription is £1 11s. 6d., payable to Hon. Treasurer.

---

*Extracts from Journal By-laws.*

4.—The *Journal* shall be delivered free to Subscribers and also to Members of the Society whose subscriptions are not in arrear.

6.—The Annual Subscription to the *Journal* is 10/6, payable to Hon. Treasurer.

---

Communications intended for insertion in the *Journal* should be sent to the Editor, Dr. J. A. Nixon, 7 Lansdown Place, Clifton, Bristol.

Books for Review and Exchange Journals should be sent to the Assistant-Editor, *Bristol Medico-Chirurgical Journal*, Medical Library, The University, Bristol.

Communications referring to the delivery of the *Journal* should be sent to the Editorial Secretary, A. L. Flemming, 48 Pembroke Road, Clifton, Bristol.

Cases for Binding Vols. I—XLVII are now ready, and may be obtained price 1/9 each (postage extra), upon application to J. W. Arrowsmith Ltd., Quay Street, Bristol; or the *Journal* will be bound, including Case, for 7/- per volume.

### Bristol Medico-Chirurgical Society.

### PAST PRESIDENTS.

1874—75. FREDERICK BRITTAN, M.D., B.A. Dub.
1875—76. ROBERT WILLIAM COE.
1876—77. SAMUEL MARTYN, M.D. Ed.
1877—78. AUGUSTIN PRICHARD, M.D. Berlin.
1878—79. HENRY EDWARD FRIPP, M.D. Würzburg.
1879—80. WILLIAM MICHELL CLARKE.
1880—81. JOSEPH GRIFFITHS SWAYNE, M.D. Lond.
1881—82. EDWARD LONG FOX, M.D. Oxon.
1882—83. JAMES GEORGE DAVEY, M.D. St. And.
1883—84. WILLIAM JOHNSTONE FYFFE, M.D., B.A. Dub.
1884—85. GEORGE FORSTER BURDER, M.D. Aberd.
1885—86. WILLIAM HENRY SPENCER, M.D., M.A. Cantab.
1886—87. FRANCIS POOLE LANSDOWN.
1887—88. LEMUEL MATTHEWS GRIFFITHS.
1888—89. ROBERT SHINGLETON SMITH, M.D., B.Sc. Lond.
1889—90. NELSON CONGREVE DOBSON, Ch.M. Brist.
1890—91. SAMUEL HENRY SWAYNE.
1891—92. FRANCIS RICHARDSON CROSS, M.B. Lond., LL.D. Brist.
1892—93. EDWARD MARKHAM SKERRITT, M.D., B.S., B.A. Lond.
1893—94. JAMES GREIG SMITH, M.B., C.M., M.A. Aberd., F.R.S.E.
1894—95. ALFRED JAMES HARRISON, M.B. Lond.
1895—96. ARTHUR WILLIAM PRICHARD.
1896—97. ALFRED EDWARD AUST LAWRENCE, M.D., C.M. Aberd.
1897—98. JOHN EDWARD SHAW, M.B., C.M. Ed.
1898—99. ROBERT ROXBURGH, M.B. Ed.
1899—00. WILLIAM HENRY HARSANT.
1900—01. DAVID SAMUEL DAVIES, M.D. Lond.
1901—02. BARCLAY JOSIAH BARON, M.B., C.M. Ed.
1902—03. GEORGE MUNRO SMITH, M.D. Brist.
1903—04. JAMES PAUL BUSH, C.M.G., C.B.E., Ch.M. Brist.
1904—05. REGINALD EAGER, M.D. Lond.
1905—06. JOHN DACRE.
1906—07. JAMES TAYLOR.
1907—08. HENRY WALDO, M.D., C.M. Aberd.
1908—09. JOHN MICHELL CLARKE, M.D , M.A. Cantab.
1909—10. JAMES SWAIN, C.B., C.B.E., M.D., M.S. Lond.
1910—11. BERTRAM MITFORD HERON ROGERS, M.D., B.Ch., B.A. Oxon.
1911—12. CHARLES ALEXANDER MORTON.
1912—13. WALTER CARLESS SWAYNE, M.D., B.S. Lond. and Brist.
1913—14. PATRICK WATSON-WILLIAMS, M.D. Lond.
1914—15. WILLIAM HARRY CHRISTOPHER NEWNHAM, M A., M.B. Cantab.
1915—16 GEORGE PARKER, M.A., M.D. Cantab.
1916—17 FRANCIS HENRY EDGEWORTH, M.A., M.D. Cantab., D.Sc. Lond.
1917—18 FREDERICK LACE.
1918—19 ROBERT GUTHRIE POOLE LANSDOWN, M.D., B.S. Durh.
1919—20 I. WALKER HALL, M.D., Ch.B. Vict.
1920—21 LEOPOLD ERNEST VALENTINE EVERY-CLAYTON, M.D. Lond.
1921—22 CYRIL HUTCHINSON WALKER, M.B., B.A. Cantab.
1922—23 JOHN ALEXANDER NIXON, C.M.G., B.A., M.D. Cantab.
1923—24 JOHN LACY FIRTH, M.D., M.S Lond.
1924—25 JOHN ODERY SYMES, M.D. Lond.
1925—26 THOMAS CARWARDINE, M.S. Lond.
1926—27 ARTHUR LAUNCELOT FLEMMING, M.B., Ch.B. Brist.
1927—28 WALTER KENNETH WILLS, M.A., M.B., B.Ch. Cantab.
1928—29 HENRY LAWRENCE ORMEROD, M.D., B.Ch. R.U.I.
1929—30 CHARLES FERRIER WALTERS.

### 1931.

### LIST OF SUBSCRIBERS

TO THE

### Bristol Medico-Chirurgical Journal.

### HONORARY MEMBERS OF THE SOCIETY.

| | |
|---|---|
| SWAIN, JAMES, *C.B.*, *C.B.E.*, M.D., M.S. Lond. .. .. .. .. | Wyndham House, Easton-in-Gordano. |
| WATSON-WILLIAMS, P., M.D.Lond. | 2 Rodney Place, Clifton. |
| SMITH, W. A., M.A., M.B. Oxon. | 70 Pembroke Road, Clifton. |

### SUBSCRIBERS.

*Those marked * are Members of the Society.*

| | |
|---|---|
| *ADAMS, A. W., M.S. Lond. ... ... ... ... ... | Rodney House, Clifton. |
| ADYE, W. J. H. ... ... ... ... ... ... ... ... | St. Margaret's, Bradford-on-Avon. |
| *ALDWINCKLE, H. M. M.B., Ch.B. Brist. ... ... | 30 E Berkeley Square, Clifton. |
| *ALEXANDER, D. A., M.B., Ch.B. Vict. ... ... | 112 Pembroke Road, Clifton, Bristol. |
| ALEXANDER, G. L., B.A. Cantab. ... ... ... | c/o Principal Medical Officer, Freetown, Sierra Leone. |
| *ALFORD, A. F., M.B., Ch.B. Brist. ... ... ... | 84 Queens Road, Bristol. |
| ALFORD, H. J., M.D. Lond.... ... ... ... ... | 4 Park Terrace, Taunton. |
| *ARMSTRONG, JEAN, M.B., Ch.B. Brist. ... ... | Ryecroft, Bell Barn Road, Stoke Bishop, Bristol. |
| *AUBREY, T., M.B. Lond. ... ... ... ... ... | Bitton, near Bristol. |
| *BAILEY, HAMILTON... ... ... ... ... ... ... ... | 240 Hendon Way, London, N. |
| *BAKER, LILY A., M.D., B.Ch., B.A.O., R.U.I. ... | Vyvyan House, Clifton. |
| *BARBER, M. C., M.B., Ch.B. Brist. ... ... ... | Ingledene, High Street, Staple Hill, Bristol. |
| *BARKER, G. H., M.D., B.Sc. Lond. ... ... ... | 124 Redland Road, Bristol. |
| *BARTHOLOMEW, E. U. ... ... ... ... ... ... ... | 12 Lansdown Place, Clifton. |
| *BATT, J. C., M.B., Ch.B. Brist. ... ... ... | Maudsley Hospital, London, S.W. |
| BEATSON, D. H., M.B., Ch.B. Brist. ... ... ... | 8 Richmond Park Road, Clifton. |
| *BENSON, ELIZABETH, M.B., Ch.B. Brist. ... ... | 8 Theresa Place, Bristol Road, Gloucester. |
| *BERGIN, F. G. ... ... ... ... ... ... ... ... | 31 Oakfield Road, Clifton. |
| BERNARD, C. ... ... ... ... ... ... ... ... ... | 564 Fishponds Road, Fishponds, Bristol. |
| *BERRILL, T. H., M.B., Ch.B. Brist. ... ... ... | Royal Northern Hospital, London, N.7. |
| BERRY, F. C., M.D., B.Ch., B.A. Dub. ... ... | Locarno, Burnham, Somerset. |
| *BERRY, R. J. A., Prof., M.D. ... ... ... ... | Director of Medical Services, Stoke Park Colony, Bristol. |
| *BINNS, R. T., M.B., B.S. ... ... ... ... ... | Combehaven, Hanham. |
| *BIRRELL, J. A., M.D., M.S. Lond. ... ... ... | 2 Dundonald Road, Redland, Bristol. |
| *BLACHFORD, J. V., *C.B.E.*, M.D. Durh. ... ... | Milverton House, Long Ashton. |
| BLACKETT, J. F., M.D., B.S. Lond. ... ... ... | Hamsterley, Bloomfield Park, Bath. |
| *BLAGG, ARTHUR F., M.D. Durh.... ... ... ... | 6 Upper Belgrave Road, Clifton. |
| *BLATHWAYT, A. de V. ... ... ... ... ... ... | 6 Brock Street, Bath. |
| BLETCHLY, G. PLAYNE, M.B. Lond. ... ... ... | Hazelwood, Nailsworth, Glos. |
| *BODMAN, A. P., M.B., Ch.B. ... ... ... ... | 11 Hurle Crescent, Clifton, Bristol. |
| *BODMAN, C. O., M.D. Durh. ... ... ... ... | 17 Upper Belgrave Road, Clifton. |
| *BODMAN, F. H., M.B., Ch.B. Brist. ... ... ... | 2 Redland Court Road, Bristol. |
| *BODMAN, J. HERVEY, M.D. Lond. ... ... ... | 11 Hurle Crescent, Clifton, Bristol. |
| *BOULTON, B. J., M.B., Ch.B. Brist. ... ... ... | Ham Green Hospital. |
| *BRADBEER, E. G., M.B., Ch.B. Brist. ... ... | 32 Linden Road, Westbury Park, Bristol. |
| *BRADLEY, W. H., B.M., B.Ch. Oxon. ... ... | St. Wulstan's, Stratton-on-Fosse, Bath. |
| *BRAY, G., Lt.-Col., *D.S.O* ... ... ... ... ... | 46 Henleaze Avenue, Westbury-on-Trym. |
| *BRENTNALL, T. C. ... ... ... ... ... ... ... | Bellfield, Brightlingsea, Essex. |
| *BRIERLEY, C. E. ... ... ... ... ... ... ... | Great Totton, Hants. |

*Brinckman, W. P., M.B., Ch.B. Brist. ... ... 4 Oakland Road, Redland, Bristol.
*Bristowe, H. C., M.D. Lond. ... ... ... ... 3 Upper Belgrave Road, Clifton.
*Brittain, H. A., M.B., Ch.B. Dub. ... ... ... "Kilronan" Anglesea Road, Dublin, Ireland.
*Britton, R. B., M.B., B.S. Lond. ... ... ... 33a Whiteladies Road, Clifton.
*Brocklehurst, R. J., M.A., D.M. Oxon. ... Bristol University.
*Buckmaster, G. A., M.A., M.D. Oxon. ... ... 8 Victoria Square, Clifton.
*Burdon-Cooper, J., M.D., B.Sc. Durh. ... ... 12 The Circus, Bath.
*Burges, R. ... ... ... ... ... ... ... ... Druid's Mead, Stoke Bishop, Bristol.
*Burges, C. P., M.B., Ch.B. ... ... ... ... Ham Green Hospital, Pill, Nr. Bristol.
*Burgess, N. F. C., M.D. Cantab.... ... ... ... Hereford House, Clifton.
*Burnett, R. H., M.B., B.S. Durh. ... ... ... 479 Bath Road, Brislington, Bristol.
*Bush, G. B., M.B., Ch.B. Brist. ... ... ... 5 Lansdown Place, Clifton.

*Cairns, F. J. ... ... ... ... ... ... ... ... Devon House, Whitehall Road, Bristol.
*Carey, R. S., *O.B.E.*, B.A. Cantab. ... ... 502 Bath Road, Brislington, Bristol.
*Carleton, H. H., M.A., M.D., B.Ch. Oxon. ... 1 Lansdown Road, Clifton.
*Carling, A., M.A., M.B., B.C. Cantab. ... ... 12 Great George Street, Bristol.
*Carwardine, Thomas, M.B., M.S. Lond. ... ... 16 Victoria Square, Clifton.
*Casson, E,, M.B., Ch.B. Brist. ... ... ... ... Dorset House, Clifton Down, Bristol.
*Cates, H. J., M.D. Lond. ... ... ... ... ... Northwoods House, Winterbourne, Bristol.
*Cave, Edward J., M.D. Lond. ... ... ... ... 16 The Circus, Bath.
*Chambers, E. R. ... ... ... ... ... ... ... 9 Clifton Park, Clifton.
*Charles, J. R., M.A., M.D., B.C. Cantab. ... 5 Rodney Place, Clifton.
*Chesterman, J. T., M.D., B.S. ... ... ... ... Glen Avon, Broomfield Road, Bath.
*Chitty, H., M.S. Lond. ... ... ... ... ... 46 Pembroke Road, Clifton.
*Christofferson, P. E., M.B., Ch.B. Brist. ... 8 Lansdown Place, Clifton.
*Clarke, R. C., *O.B.E.*, M.B., Ch.B. Brist. ... 29 Victoria Square, Clifton.
*Coates, Vincent M., *M.C.*, M.A., M.D., B.C. Cantab. ... ... ... ... ... ... 10 The Circus, Bath.
Connellan, P. S. ... ... ... ... ... ... ... 12 Bloomsbury Street, London, W.1.
*Cookson, R. G. ... ... ... ... ... ... ... 35 Pembroke Road, Bristol.
*Coombs, Carey, M.D. Lond. ... ... ... ... 3 Pembroke Road, Clifton.
*Cooper, William Russell ... ... ... ... ... 13 Westfield Park, Redland, Bristol.
*Corfield, C., M.D. Durh. ... ... ... ... ... 5 Kensington Place, Clifton.
*Cornall, A. F. M. ... ... ... ... ... ... Carnarvon Lodge, Redland, Bristol.
*Cory, R. A. S. ... ... ... ... ... ... ... Montego Bay, Jamaica, B.W.I.
*Cossham, W. L., M.B., Ch.B. Brist. ... ... 32 Somerville Road, St. Andrew's Park, Bristol.
*Courtney, R. M., M.B., Ch.B. Glasg. ... ... 487 Gloucester Road, Bristol.
*Coxon, Beatrice .. ... ... ... ... ... ... 16 Richmond Hill, Clifton.
Cridland, A. B. ... ... ... ... ... ... ... Salisbury House, Chapel Ash, Wolverhampton.
*Cross, F. R., M.B. Lond., LL.D. Brist. ... ... Worcester House, Clifton.
*Crossman, F. W., M.B. Durh. ... ... ... ... White's Hill, Hambrook, near Bristol.

*Dacre, John ... ... ... ... ... ... ... ... 14 Eaton Crescent, Clifton.
*Datta, S. S. M.B., Ch.B. Brist. ... ... ... ... Snowdon House, Fishponds, Bristol.
*Davies, E. D. D. ... ... ... ... ... ... ... Standish House, Stonehouse, Glos.
Devine, H., *O.B.E.*, M.D. Lond. ... ... ... Holloway Sanatorium, Virginia Water.
*Dawe, J. H., M.B. Edin. ... ... ... ... ... Lynwood House, Bickford Road, Bath.
*Dawson, W. R., *O.B.E.*, M.D. Dub.... ... ... 18 Brock Street, Bath.
*Dix, C. ... ... ... ... ... ... ... ... ... 11 Victoria Square, Clifton.
*Dixon, Helen M., M.B., B.Ch. Brist. ... ... 10 Whatley Road, Clifton.
*Dixon, T. B. ... ... ... ... ... ... ... ... 11 Pembroke Road, Clifton, Bristol.
*Drapes, T. L., M.B., B.C., B.A. Cantab. ... St. Maur, Beaufort Square, Chepstow.
*Duerden, J. R., M.B., Ch.B. Brist. ... ... 149 Bell Hill Road, St. George, Bristol.
*Dutton, Hilda R. ... ... ... ... ... ... 405 Stapleton Road, Bristol.

*Eglinton, J. H. C. ... ... ... ... ... ... Street, Somerset.
*Elkington, G. W., M.B., B.S. Lond. ... ... Malvern College, Malvern.
Elliot Bros, Ltd.... ... ... ... ... ... ... 20-22 O'Connell Street, Sydney, N.S.W. Australia.

| | |
|---|---|
| Elliott, F. Percy, M.B., C.M. Aberd. | 113 Grove Rd., Walthamstow, London, E.17. |
| *Elliott, W. H. A., M.B., B.S. Lond. | 116 Pembroke Road, Clifton. |
| *Every-Clayton, L. E. V., M.D. Lond. | Dunkery Lodge, Minehead, Som. |
| *Faill, C. J. C. | 19 Portland Square, Bristol. |
| *Fallon, Col. J. | 35 Henleaze Gardens, Henleaze, Bristol. |
| *Faraker, E. C. | 85 Coldharbour Road, Westbury Park, Bristol. |
| *Farquharson, J. L. | Breda, Weston-super-Mare. |
| Farnfield, W. W. | Clive Vale, Gillingham, Dorset. |
| *Fawn, G. F. | 6 Oakfield Road, Clifton. |
| *Fayle, B. W. D., M.B., Ch.B. | Reine Barnes, Dursley, Glos. |
| *Fells, A., M.B., C.M. Edin. | 6 Elton Road, Clifton. |
| *Fells, G. R., M.B., Ch.B. Brist. | Almondsbury. |
| *Fells, R. R., M.B., B.S. Lond. | 12*d* Cotham Road, Bristol. |
| *Feneley, G. L., M.B., Ch.B. | Englewood, Stoke Lane, Westbury-on-Trym. |
| *Firth, J. L., M.D., M.S. Lond. | 8 Victoria Square, Clifton. |
| *Fisher, A. C. | Kalene Hill, Mwini Lunga, N.W. Rhodesia. |
| *Flemming, A. A. G. | Bristol Royal Infirmary. |
| *Flemming, A. L., M.B., Ch.B. Brist. | 48 Pembroke Road, Clifton. |
| *Flemming, C. E. S. | Manvers House, Bradford-on-Avon. |
| *Forrester-Brown, Maud, M.D., M.S. Lond. | 22 Coombe Park, Bath. |
| *Foss, E. V., M.D., Ch.B. Brist. | Summer Hill House, St. George, Bristol. |
| *Fox, F. E., B.A. Camb. | Brislington House, Brislington, Bristol. |
| *Fraser, A. D., M.A., M.B., Ch.B. Glasg. | 4 Alexandra Road, Clifton. |
| *Fraser, D., M.B., Ch.B. Edin. | Westgate, Dursley, Glos. |
| *Freeth, H., M.D. Dub. | 93 Redland Road, Bristol. |
| *Fuller, H. L. | 9 Gay Street, Bath. |
| *Garden, R. R., M.A., M.B., B.Ch. Aberd. | 9 Harley Place, Clifton. |
| Garland, E. B., M.B., C.M. Edin. | 114*a* Hampton Road, Redland, Bristol. |
| *Gee, C. A. H., M.B., Ch.B. Brist. | Portbury, Nr. Bristol. |
| *Gibbs, A. N. Godby | 30 Whiteladies Road, Clifton. |
| *Giraldi, J. J., M.B., Ch.B. Brist. | Gibraltar. |
| *Glenny, E. T., M.B., B.S. Lond. | 102 Pembroke Road, Clifton. |
| *Gordon, R. G., M.D., B.Sc. Edin. | 9 The Circus, Bath. |
| *Gorham, A. P., M.B., Ch.B. Brist. | 53 Westbury Road, Westbury-on-Trym. |
| *Gornall, W. A., M.B., Ch.B. Brist. | Orchard House, Nailsea, Somerset. |
| *Green, T. A., *D.S.O.*, M.D., C.M. Edin. | 33*a* Whiteladies Road, Clifton. |
| *Greenlaw, Madge E., M.B., Ch.B. Brist. | 194 Redland Road, Bristol. |
| Griffiths, Cornelius A. | 35 Newport Road, Cardiff. |
| *Griffiths, J. S. | Redland Park House, Bristol. |
| *Griffiths, S. J. H., M.B., Ch.B. Brist. | 1 Victoria Square, Clifton. |
| *Groves, E. W. Hey, M.D., M.S. Lond. | 25 Victoria Square, Clifton. |
| *Hadfield, G., M.D. Lond. | Royal Free Hospital, London. |
| *Hall, E. George, M.B. Lond. | 42 Apsley Road, Clifton, Bristol. |
| *Hall, I. Walker, M.D., Ch.B. Vict. | Shortwood Lodge, Pucklechurch, Glos. |
| *Ham, C. I., M.B., Ch.B. Brist. | 16 Elm Lane, Redland, Bristol. |
| *Harris, H. Elwin, B.A., M.B. Cantab. | 13 Lansdown Place, Clifton |
| *Harris, H. E., M.A., M.B., B.Ch. Cantab. | 13 Lansdown Place, Clifton. |
| *Harsant, W. H. | Tower House, Clifton Down Road, Clifton. |
| *Hartley, G., M.B., B.S Lond. | 28 Pembroke Road, Clifton. |
| *Henderson, James, M.B., Ch.B. Aberd. | Nordrach-upon-Mendip, Blagdon. |
| *Hector, F., M.D. Lond. | Chelsea Hospital for Women, Arthur Street, Chelsea, S.W.3. |
| *Herapath, C. E. K., *M.C.*, M.D. Lond. | Ormlie, Clifton. |
| *Heron, Gordon, M.B., Ch.B. Brist. | 34 Gloucester Road, Bristol. |
| *Heron, Gordon, Mrs., M.B., Ch.B. Brist. | 34 Gloucester Road, Bristol. |
| *Hooker, J. A., M.B., Ch.B. Brist. | 16 St. Edyth's Road, Sea Mills. |
| *Hosken, J. G. F. | Uley, Glos. |

Hospital, Bristol General (*Secretary*, T. W. Gregg).
*Houghton, Lydia M., M.B., B.S. Lond. ... ... 74 Church Road, Redfield, Bristol.
*Hughes, T. I. ... ... ... ... ... ... ... ... Bristol General Hospital.
*Hughes F. W. Terrell, M.B., Ch.B. Brist. ... Chew Magna, Somerset.
*Hunter, F. S. ... ... ... ... ... ... ... ... c/o Messrs. J. Wright & Sons Ltd., Publishers, Bristol.

*Iles, A. E., *O.B.E.*, M.B., B.S. Lond. ... ... 17 Victoria Square, Clifton.
Infirmary, Bristol Royal (*Secretary*, E. C. Smith).
Ingle, C. Durban ... ... ... ... ... ... Somerton, Somerset.

*Jackman, W. A., M.B., Ch.B. Brist.... ... ... 15 Mortimer Road, Clifton.
*James, J. A., M.D. Lond. ... ... ... ... ... 5 Rodney Place, Clifton.
*James, April D., M.B., Ch.B. Brist. ... ... Eye Hospital, Birmingham.
*Jenkins, R. D. ... ... ... ... ... ... ... ... Almondsbury.
*Jenkins, F. G., M.B., Ch.B. Brist. ... ... ... 51 Redcliff Hill, Bristol.
*Johnson, R. G., M.B. Lond. ... ... ... ... 119 Redland Road, Bristol.
*Joll, C. A., M.B., M.S. Lond. ... ... ... ... 64 Harley Street, London.
*Joscelyne, P. C., M.B., Ch.B. Brist.... ... ... 70Longmead Avenue, Bishopston, Bristol.

*Kemm, N. F., M.B., B.S. Lond. ... ... ... 188 Redland Road, Bristol.
*Kennedy, W. P., M.D. Dub. ... ... ... ... 3 Gay Street, Bath.
*Kerfoot, Stanley J. ... ... ... ... ... ... ... 194 Redland Road, Bristol.
*Kingston, S. H., M.B., Ch.B. Brist. ... ... 3 Whiteladies Road, Clifton.
*Kyle, H. G., M.A., M.D., B.Ch. Oxon. ... ... 31 Westbury Road, Bristol.

*Lace, F. ... ... ... ... ... ... ... ... ... ... 5 Gay Street, Bath.
*Lancaster, L. C., B.A. Cantab. ... ... ... Bristol General Hospital.
*Langley, G. F., M.B., Ch.B. Brist. ... ... ... Royal Free Hospital, London, W.C.
*Lees, E. Leonard, M.D., C.M. Edin. ... ... 22 Richmond Hill, Clifton.
*Legat, R. Eddowes, M.B., C.M. Edin. ... ... Murray House, Staple Hill, Bristol.
Leigh, W. W. ... ... ... ... ... ... ... ... Llansamnor House, Cowbridge, Glam.
*Leonard, R. C., M.D. Durh. ... ... ... ... Bower Ashton, Bristol.
*Lester, Muriel A., M.B., B.S. ... ... ... 110 Avon Vale Road, Redfield, Bristol.
*Levis, J. S., *M.C.*, M.B., B.Ch., N.U.I. ... ... 20 Gay Street, Bath.
*Linton, Marion S., M.B. Lond. ... ... ... 21 Oakfield Road, Clifton.
*Lister, A. E. J., M.B., B.S. Lond. ... ... ... 86 Pembroke Road, Clifton.
*Lister, L. Margaret ... ... ... ... ... ... 12 All Saints Road, Clifton.
*Logan, H. B. ... ... ... ... ... ... ... ... ... Elmwood, Aberdeen Road, Clifton.
*Lucas, R. P., M.B., Ch.B. Brist. ... ... ... Bristol Royal Infirmary.
*Lucas, J. E. ... ... ... ... ... ... ... ... ... 48 Coronation Road, Bristol.
*Lucas, J. J. S., M.D. Lond. ... ... ... ... 186 Wells Road, Totterdown, Bristol.
*Lysaght, A. C. ... ... ... ... ... ... ... 8 Clifton Hill, Bristol.

*Macfie, J. D., M.B., Ch.B. Glasg. ... ... ... Winsley Sanatorium, near Bath.
Mackinnon, Charles, M.B., C.M., M.A. Glasg. Davaar, Cirencester.
Maclean, Sir Ewen J., M.D., C.M. Edin. ... 12 Park Place, Cardiff.
*MacLeod, Donald, M.B., C.M. Edin. ... ... 84 Redland Road, Redland, Bristol.
*MacLeod, G., *M.C.*, M.D., Ch.B. ... ... ... "Wargrave," Clevedon.
*MacWatters, J. C., *M.B.E.* ... ... ... ... Almondsbury, near Bristol.
Marle, S. ... ... ... ... ... ... ... ... Newbridge Road, Bath.
*Marshall, A. H., M.B., Ch.B. Bristol. ... ... Odelos, Canford Lane, Bristol.
Marshall, Arthur L., M.B., B.A. Cantab. ... Raveningham, Norwich.
*Martin, P. S. ... ... ... ... ... ... ... Victoria Quadrant, Weston-super-Mare.
Mather, J. S., M.B., C.M. Aberd. ... ... ... Lawrence Hill House, Bristol.
*Mayes, F. J. A. ... ... ... ... ... ... ... ... 79 Pembroke Road, Clifton.
*McCrea, Phillip, M.D., B.Ch. Dub. ... ... ... 56 Kellaway Avenue, Bristol.
*McKenzie, W. G., *M.C.* ... ... ... ... ... 101 Coronation Road, Bristol.
*McLannahan, J. G. ... ... ... ... ... ... The Mount, Stonehouse.
*Meadows, G. ... ... ... ... ... ... ... ... ... Auteuil, Southmead Road, Bristol.
Miall, C. L'O. ... ... ... ... ... ... ... ... Elm Hayes, Paulton, near Bristol.
*Milling, T., M.B., Ch.B. Belfast ... ... ... 1 Vicarage Road, Southville, Bristol.

| | |
|---|---|
| *Milner, A., M.B., B.Ch. Dub. ... ... ... | 61 Cotham Brow, Bristol. |
| *Moore, R. M., M.A., M.B., Ch.B. Cantab. ... | Mundens, Malmesbury, Wilts. |
| *Moore, C. A., M.B., M.S. Lond. ... ... ... | 56 St. Paul's Road, Clifton. |
| *Moore, L. A. ... ... ... ... ... ... ... ... | Hillsborough, Cotham Park, Bristol. |
| *Morris, A. G. ... ... ... ... ... ... ... ... | 18 Westbury Park, Westbury-on-Trym. |
| *Morris, L. N., M.B., Ch.B. Brist. ... ... ... | 24 All Hallows Rd., Upper Easton, Bristol |
| *Morison, A. G., M.A., M.B., Ch.B., D.P.H. ... | 34 Woodstock Road, Redland, Bristol. |
| *Mumford, W. G., M.B. Lond. ... ... ... ... | 18 The Circus, Bath. |
| *Mundy, G. S., M.B., B.Ch. Brist. ... ... ... | Homewood, Coombe Dingle, Bristol. |
| *Murphy, F. D., M.B., Ch.B., B.Sc. Cork ... | |
| *Myles, G. T. ... ... ... ... ... ... ... ... | 7 Apsley Road, Clifton. |
| *Myles, Q. St. L., M.A., B.M., B.Ch. Oxon. | 7 Apsley Road, Clifton. |
| *Neild, Newman, M.B., B.Ch. Vict. ... ... ... | 9 Richmond Hill, Clifton. |
| *Newnham, W. H. C., M.B., M.A. Cantab. ... | 3 Lansdown Place, Victoria Square, Clifton. |
| *Nichols. F. C., *M.C.*, M.B., Ch.B. Brist. ... | 38 Whiteladies Road, Clifton. |
| *Nixon, J. A., *C.M.G.*, M.D. Cantab. ... ... | 7 Lansdown Place, Clifton. |
| *Noble, J. Innes, M.B., Ch.B. Liverp. ... ... | 102 Pembroke Road, Clifton. |
| *Nott, Winifred, M.B., Ch.B. Brist.... ... ... | "Fassiefern," Upper Cranbrook Road, Redland, Bristol. |
| *O'Brien, C., M.B., Ch.B. ... ... ... ... ... | Bristol Children's Hospital. |
| *Ormerod, G. L., B.A. Cantab. ... ... ... ... | 2 Henleaze Road. |
| *Ormerod, H. Lawrence, M.D., B.Ch. R.U.I. ... | 2 Henleaze Road, Westbury-on-Trym, Bristol. |
| Orr-Ewing, H. J., *M.C.*, M.D. Lond. ... ... | English Mission Hospital, Jerusalem. |
| *Parker, George, M.A., M.D. Cantab. ... ... | 9 Pembroke Road, Clifton. |
| *Parkes, J. A., M.B., Ch.B. Liverp. ... ... ... | 8 South Road, Redland, Bristol. |
| Parkinson, Lt.-Col. G. S., *D.S.O.*, R.A.M.C. ... | R.A.M.C. Mess, Grosvenor Place, London, S.W.1. |
| *Parry, R. H., M.B., B.S. Lond. ... ... ... | Newby, Holmes Grove Road, Henleaze. |
| Parsons, Sir J. H., M.B., B.S., B.Sc. Lond. ... | 54 Queen Anne Street, London, W. |
| *Pearce, J. L., M.B., Ch.B. Edin. ... ... ... | Hambrook Court, near Bristol. |
| *Perry, Bruce, M.B., Ch.B. Brist. ... ... ... | 1 Berkeley Square, Clifton. |
| *Phillips, P., M.B., Ch.B. Brist.... ... ... ... | Southmead Hospital, Bristol. |
| *Pinkerton, J. M., B.Sc. Wales; M.B., Ch.B. Glasg. | 5 The Circus, Bath. |
| *Pollard, J., M.B., B.Ch., B.A.O., N.U.I. ... | 88 Coronation Road, Bristol. |
| *Potter, Mabel F., M.B., Ch.B. ... ... ... | Bristol Royal Infirmary. |
| *Powell, H. F., M.D. Brux. ... ... ... ... | Ellenborough Park, Weston-super-Mare. |
| *Powell, J. J., M.D. Lond. ... ... ... ... | 2 Gloucester Road, Bishopston, Bristol. |
| *Price, A., M.B., Ch.B. Brist. ... ... ... ... | Bristol General Hospital. |
| *Price, N. L., M.B., Ch.B. Brist. ... ... ... ... | Bristol General Hospital. |
| *Pring, C. E. ... ... ... ... ... ... ... ... | 14 West Park, Clifton, Bristol. |
| *Prowse, D. C., M.B., Ch.B. Brist. ... ... ... | Wigmore House, Thornbury, nr. Bristol. |
| *Rayner, D. C., Ch.M. Brist. ... ... ... ... | 9 Lansdown Place, Clifton, Bristol. |
| *Renton, R. A. S., *M.C.*, M.D. Durh. ... ... | Emsworth House, Clevedon. |
| *Robertson, D., M.B., Ch.B. Edin. ... ... ... | 205 Wells Road, Knowle, Bristol. |
| *Rogers, Herbert, M.B., Ch.B. Brist. ... ... | 11 Clifton Hill, Bristol. |
| *Rolfe, R., B.A., M.B., B.C. Cantab. ... ... | Park House, St. Andrew's Road, Avonmouth. |
| *Ross, C. C., M.D. Man.... ... ... ... ... ... | Minnedosa, Manitoba, Canada. |
| *Rowley, A. T. ... ... ... ... ... ... ... ... | Wrington. |
| *Salisbury, Walter, M.D., M.S. Lond. ... ... | Woodlands, 20 Billing Road, Northampton. |
| Savage, W. G., M.D., B.Sc. Lond. ... ... ... | "Bourn," 7 Trewartha Park, Weston-super-Mare. |
| *Scarff, G., M.B., Ch.B. Edin. ... ... ... ... | 5 Rodney Place, Clifton. |
| *Scott, H. H. ... ... ... ... ... ... ... ... | 78 Old Market Street, Bristol. |
| *Shaw, J. E., M.B., C.M. Edin. ... ... ... ... | 23 Caledonia Place, Clifton. |
| *Shepherd, H. L., M.B., Ch.M. Brist. ... ... | 16 Victoria Square, Clifton. |

| | |
|---|---|
| *Short, A. R., B.Sc., M.D., B.S. Lond. ... ... | 69 Pembroke Road, Clifton. |
| *Short, L. J., M.D., B.S. Lond., M.D. Brist. ... | Maywood, Atlantic Road South, Weston-super-Mare. |
| *Smith, G. Cockburn, M.D. Brux. ... ... ... | 12 South Road, Newton Abbot. |
| *Smith, T. Wilson, M.D. Lond. ... ... ... ... | 26 The Circus, Bath. |
| *Smythe, H. J. D., *M.C.*, M.B., M.S. Lond. ... | 15 Eaton Crescent, Clifton. |
| *Somers, Mary F. E. ... ... ... ... ... ... | 2 Ashcombe Road, Weston-super-Mare |
| *Spoor, A., M.B., B.Ch. Cantab. ... ... ... | The Hydro, Bristol. |
| *Staniland, M. F. ... ... ... ... ... ... ... | 255 Stapleton Road, Bristol. |
| *Statham, R. S. S., *O.B.E.*, M.D., M.Ch. Brist. | 10 Beaufort Road, Clifton, Bristol. |
| *Stewart, A. L., D.M., D.Ch. Brux. ... ... | 31 Howard Road, Southville, Bristol. |
| *Stock, W. S. V., M.B. Lond. ... ... ... ... | 1 Mortimer Road, Clifton. |
| *Stone, D. M., M.D., B.S. Lond. ... ... ... ... | 38 Upper Belgrave Road, Durdham Down, Bristol. |
| *Strover, H. W. M., *O.B.E.*, M.B., Ch.B. Aberd. ... ... ... ... ... ... ... | 24 Gloucester Road, Bishopston, Bristol. |
| *Sutherland, Gordon ... ... ... ... ... ... | 5 Rodney Place, Clifton, Bristol. |
| *Sutton, G. E. F., M.B., B.S. Lond. ... ... ... | 1 Pembroke Road, Clifton. |
| *Symes, J. O., M.D. Lond. ... ... ... ... ... | 71 Pembroke Road, Clifton. |
| *Tasker, D. G. C., M.B., M.S. Lond. ... ... | 10 Christchurch Road, Clifton. |
| *Taylor, A. L., M.D. Lond. ... ... ... ... ... | Bristol General Hospital. |
| Taylor, A. L. ... ... ... ... ... ... ... ... | Bristol Royal Infirmary. |
| *Taylor, H. N., M.A. Cantab., M.D. Dub. ... | Hillside, Ebbw Vale. |
| *Temple, G. H., M.B., C.M. Edin. ... ... ... | Ailanthus, Weston-super-Mare. |
| *Terry, C. H., M.A., M.B., B.Ch. Oxon. ... ... | 15 The Circus, Bath. |
| Thin, James ... ... ... ... ... ... ... ... | 54 & 55 South Bridge, Edinburgh. |
| *Thompson, J., M.B., Ch.B. Edin. ... ... ... | Eastbourne House, Devizes. |
| *Thomas, J. D., M.B. Cantab. ... ... ... ... | 50 Belvoir Road, St. Andrews, Bristol. |
| *Todd, A. T., *O.B.E.*, M.B., Ch.B. Brist. ... ... | 10 Tyndall's Park, Clifton. |
| *Tripp, Dorothy, M.H., M.B., Ch.B. Brist. ... | Winthill, Banwell, Som. |
| *Trist, J. R. R., *M.C.* ... ... ... ... ... | 120 Henleaze Road, Henleaze. |
| *Tryon, Victoria S., M.B., Ch.B. Brist. ... ... | 3 Harley Place, Clifton. |
| *Vickery, W. H. ... ... ... ... ... ... ... | "Shirley," Broadoak Road, Weston-super-Mare. |
| *Walbank, A., M.B., Ch.B. Leeds ... ... ... | Rodney House, Clifton. |
| *Walker, Cyril H., B.A., M.B. Cantab. ... ... | 8 Oakfield Road, Clifton. |
| *Walsh, M. J., M.B., Ch.B. Dub. ... ... ... ... | 2 Knowle Road, Bristol. |
| *Walters, C. F. ... ... ... ... ... ... ... | 5 Mortimer Road, Clifton. |
| *Wansborough, T. B., M.B., Ch.B. Brist. ... ... | 8 Mortimer Road, Clifton. |
| *Wanklyn-James, C. W., *O.B.E.* ... ... ... | 3 Mortimer Road, Clifton. |
| *Warren, R., M.A., M.D. Oxon. ... ... ... ... | Ellenborough Hall, Weston-super-Mare. |
| *Waterhouse, R., M.D. Lond. ... ... ... ... | 25 The Circus, Bath. |
| *Watson-Williams, E., *M.C.*, B.A., M.B., Ch.M. Cantab. ... ... ... ... ... ... ... ... | 12 Victoria Square, Clifton. |
| *Weatherhead, E., M.B., B.Ch. Cantab. ... | 115 Queen's Road, Clifton, Bristol. |
| *Weddell, C. W. ... ... ... ... ... ... ... | Boydville, 46 Miller St., West Melbourne, Australia |
| *Weston, B. A. A., M.B., B.Ch. Brist. ... ... | 30 Cotham Grove, Bristol. |
| Weston, B. A., M.B ... ... ... ... ... ... | Brookside, Admaston, Wellington, Shropshire. |
| *Whelton, A., B.A., M.B., Ch.B. Cork ... ... | Southmead Hospital. |
| *Whicher, A. H. ... ... ... ... ... ... ... | 115 Queen's Road, Clifton. |
| *White, E. B. C. ... ... ... ... ... ... ... | Mental Hospital, Fishponds, Bristol. |
| Whitehouse, H. Beckwith, M.S. Lond. ... | 62 Hagley Road, Edgbaston, Birmingham. |
| Willcox, R. Lewis ... ... ... ... ... ... | 97 London Road, Salisbury. |
| Williams, S. R., M.B. Lond. ... ... ... ... | Buckfastleigh, Devon. |
| *Williams, A. R., M.B., Ch.B. Brist. ... ... | Bristol Royal Infirmary. |
| *Wills, W. K., *O.B.E.*, M.B., B.C. Cantab. ... | 1 Victoria Square, Clifton. |
| *Wood, Duncan ... ... ... ... ... ... ... | 5 Eaton Crescent, Clifton. |
| *Wood, W. V., *M.C.*, B.A. Cantab. ... ... ... | Henley Lodge, Yatton, Som. |
| *Wright, A. J. M.B., B.S. Lond. ... ... ... | Clifton Park, Clifton. |
| *Zealand, L., M.D. Man. ... ... ... ... ... | Brecon Lodge, Westbury-on-Trym, Bristol. |

### Publications Received.

From THE BUREAU FOR INCREASING THE USE OF QUININE.
*Chinin in der Allgemeinpraxis.* By FRITZ JOHANNESSOHN. 1930.

From CASSELL & CO. LTD.:
*Radium Therapy: Principles and Practice.* By G. E. BIRKETT, M.C., B.A., M.R.C.S.

From WILLIAM HEINEMANN (MEDICAL BOOKS) LTD.:

*The Care and Nursing of the Infant.* By Miss D. A. KENNEDY, S.R.N.

From HODDER & STOUGHTON LTD.:
*Anatomy in the Living Model.* By DAVID WATERSTON, M.A., M.D., F.R.C.S.E., F.R.S.E.

From PAUL B. HOEBER:
*The Beginnings, Egypt and Assyria.* By WARREN R. DAWSON, F.R.S.E.
*Physiology.* By JOHN F. FULTON, M.D.
*Internal Medicine.* By Sir HUMPHRY ROLLESTON, Bart., G.C.V.O., K.C.B., M.D., Hon. D.SC., D.C.L., LL.D.
*Anatomy.* By GEO. W. CORNER, M.D.
*Medicine in the British Isles.* By Sir D'ARCY POWER, K.B.E., F.R.C.S.

From THE LANCET LTD.:
*Guy's Hospital Reports.* October, 1930.

From H. K. LEWIS & CO. LTD.:
*Deafness and its Alleviation by Operation.* By VINCENT NESFIELD, F.R.C.S. 2nd Edition.
*Diseases of the Tongue.* 3rd Edition. By W. G. SPENCER, M.S., F.R.C.S. and S. CADE, F.R.C.S.
*Seasonal Variation in Man.* By E. HUGHES, M.R.C.S., L.R.C.P.

From THE OXFORD UNIVERSITY PRESS:
*The Treatment of Chronic Deafness by the Electrophonoïde Method of Zünd-Burguet.* 2nd Edition. By GEORGE C. CATHCART, M.A., M.D.
*An Introduction to Pharmacology and Therapeutics.* 2nd Edition. By J. A. GUNN, M.D., D.SC.

From THE SHELDON PRESS:
*Cancer and Scientific Research.* By BARBARA HOLMES, PH.D.

From SOCIÉTÉ SCIENTIFIQUE DE BRUXELLES:

*La Pathologie du Système Pallido-Strié.* Par P. VAN GEHUCHTEN.

From THE WELLCOME FOUNDATION LTD.:
*Cinchona Tercentenary Celebration and Exhibition.* 1930.

From JOHN WRIGHT & SONS, LTD.:
*Infant Feeding in General Practice.* By J. V. C. BRAITHWAITE, M.D., M.R.C.P.
*Surgical Handicraft.* By WALTER PYE.
*The British Journal of Surgery.* Vol. XVIII. No. 71. January, 1931.

